# A Comparative Study on Physicochemical Characteristics of Raw Goat Milk Collected from Different Farms in Malaysia

**DOI:** 10.21315/tlsr2018.29.1.13

**Published:** 2018-03-02

**Authors:** Syarifah Hazirah Syd Jaafar, Roshada Hashim, Zaiton Hassan, Norlelawati Arifin

**Affiliations:** Department of Food Biotechnology, Faculty of Science and Technology, Universiti Sains Islam Malaysia, 71800 Nilai, Negeri Sembilan, Malaysia

**Keywords:** Breed, Goat Milk, Physicochemical Composition, Principal Component Analysis, Farm

## Abstract

This study was conducted to determine the physical and chemical composition of goat milk produced by eight local farms located in the central region of Malaysia. Farms 1 to 4 (F1-SC, F2-SP, F3-SP, F4-SBC) reared Saanen-type goats while farms 5 to 8 (F5-JK, F6-JPEC, F7-JTC, F8-JC), Jamnapari-type goats. The common feedstuffs used in all farms comprised of fresh or silage from Napier grass, feed pellets, and brans while two farms, F5-JK and F6-JPEC supplemented the feeds with soybean-based product. The total solid content, dry matter, and proximate composition of goat milk and feedstuffs from the different farms were determined and the results analysed using principal component analysis. Total solid content of goat milk from the Jamnapari crossbreed had the highest solid content ranging from 11.81% to 17.54% compared to milk from farms with Saanen and Saanen crossbreed (10.95% to 14.63%). Jamnapari-type goats from F5-JK, F6-JPEC, and F8-JC had significantly higher (*p* < 0.05) milk fat and protein contents (7.36%, 7.14%, and 6.59% fat; 5.08%, 6.19%, and 4.23% protein, respectively) than milk from other farms but, milk produced by Saanen-type goats from F4-SBC contained similar protein content (4.34%) to that from F8-JC. Total ash and carbohydrate contents in milk ranged between 0.67% to 0.86% and 3.26% to 4.71%, respectively, regardless of goat breed. Feeding soybean-based products appear to have a positive influence on milk fat and protein content in Jamnaparitype goats.

## Abstract (ru)

Kajian ini dijalankan untuk menentukan komposisi fizikal dan kimia susu kambing yang dihasilkan oleh lapan ladang tempatan yang terletak di kawasan tengah Malaysia. Ladang 1 hingga 4 (F1-SC, F2-SP, F3-SP, F4-SBC) menternak kambing jenis Saanen sementara ladang 5 hingga 8 (F5-JK, F6-JPEC, F7-JTC, F8-JC) menternak kambing jenis Jamnapari. Bahan makanan biasa yang digunakan di semua ladang terdiri daripada rumput segar atau silaj dari rumput Napier, feed pellets, dan bran sementara dua ladang lagi, F5-JK dan F6-JPEC menambah makanan dengan produk berasaskan kacang soya. Kandungan pepejal, bahan kering dan komposisi susu kambing serta bahan makanan dari ladang yang berbeza telah ditentukan dan keputusan dianalisis dengan menggunakan analisis komponen utama. Kandungan pepejal susu kambing dari kacukan Jamnapari mempunyai kandungan pepejal tertinggi iaitu 11.81% hingga 17.54% berbanding susu dari ladang dengan Saanen dan kacukan Saanen (10.95% hingga 14.63%). Kambing jenis Jamnapari dari F5-JK, F6-JPEC, dan F8-JC mempunyai kandungan lemak dan protein susu (*p* < 0.05) yang lebih tinggi (7.36%, 7.14% dan 6.59% protein; 5.08%, 6.19% dan 4.23% ) daripada susu dari ladang lain tetapi susu yang dihasilkan oleh kambing jenis Saanen dari F4-SBC mengandungi kandungan protein yang sama (4.34%) dengan F8-JC. Kandungan abu dan karbohidrat dalam susu adalah di antara 0.67% hingga 0.86% dan 3.26% hingga 4.71%, tanpa mengira baka kambing. Memberi makan produk berasaskan kacang soya dilihat mempunyai pengaruh positif terhadap kandungan lemak susu dan protein dalam kambing jenis Jamnapari.

## INTRODUCTION

Goat and goat milk production is a fast-growing industry and is now considered an important economic commodity in many countries. Between 1991 and 2011 goat production increased by 55% and that of goat milk was 70% (Garcia *et al*. 2014) indicating a high demand for goat, goat milk, and its products. The expansion of goat farming in the last decade is probably due to the ability of goat to provide high quality food under diverse climatic conditions and its resilience to the extreme and capricious environment (Silanikove *et al*. 2010). In Malaysia, farmed goat population was estimated to increase from 429,398 to 439,667 goats between year 2014 to 2015 (Department of Veterinary Services 2016). However, the dairy goat production is still considered as a small entity with no local breed specifically bred for milk production, and it is reported that only about 8,195 heads of dairy goats are reared in Peninsular Malaysia until year 2014 (Sithambaram & Nizam 2014).

The major nutrient composition of goat milk is comparable to cow milk as it possesses an average of 3.4% protein, 3.8% fat, 4.1% lactose, and 0.8% ash content (Park *et al*. 2007). Indeed, milk from goat has also been found to contain beneficial nutrients and is a viable alternative to cow milk as it is less allergenic and has better digestibility (Haenlein 2004; Park *et al*. 2007). In addition, goat milk contains a high proportion of medium chain fatty acids (C6:0, C8:0, and C10:0) which partly contribute to the specific “goaty” flavour of goat milk (Silanikove e*t al*. 2010). The medium chain fatty acids are also known to be antibacterial (Johny *et al*. 2009; Batovska *et al*. 2009), antiviral (Hornung *et al*. 1994; Thormar *et al*. 1987), inhibit development and dissolve cholesterol deposits (Shingfield *et al*. 2008).

Quality of milk and its composition varies according to breed, diet and feeding practices, management system, lactation stage, parity, and animal health (Park *et al*. 2007; Goetsch *et al*. 2011). In the study of milk affected by breed factor, Sung *et al*. (1999) evaluated the milk quality from four dairy goat breeds include Alpine, Nubian, Saanen, and Toggenburg which reared in Taiwan and reported that Nubian goats had a higher percentage of fat and protein content than the other three breeds. The study by Mayer and Fiechter (2012) however showed that there was no significant difference in milk composition among the six dairy breeds in Austria. This indicated that, although similar breeds were reared in several countries, the content of milk composition could vary according to the places.

For the effect of diet on milk quality, Rego *et al*. (2008) observed that the inclusion of soybean meal concentrate in the cow feed tend to increase the milk fat concentration. Besides, Aplocina and Spruzs (2012) reported that the addition of fodder yeast, sunflower cake, and wheat bran into basic feed ration significantly (*p* < 0.05) increased the fat content of goat milk and wheat bran also significantly (*p* < 0.05) increased the milk protein content. These showed that the different types of feedstuffs contribute to the different effect on the milk composition. Therefore, the different farms with different goat breeds and type of feedstuffs available will have different milk nutrient content.

Recently, Alyaqoubi *et al*. (2015) determined the physicochemical properties and antioxidant activity of milk from five different goat breeds and Lai *et al*. (2016) studied the physicochemical and microbial qualities of raw goat milk locally produced in one farm in Malaysia. However, Lai *et al*. (2016) stated there is a lack of current data regarding goat milk properties in Malaysia and more study is needed to obtain the updating data as a reference. Although numerous studies of goat milk composition and its quality affected by various factors had been done worldwide, there is a lack of local studies information regarding the nutritional composition of goat milk produced at the farm level. The study on the composition of locally produced goat milk can provide the range of the nutrient content data and can be compared with the other parts of the world as well as assessments can be made on the milk quality to extend its benefits. Therefore, the purpose of this study was to profile the composition of goat milk locally produced from different farms in Negeri Sembilan and Selangor. The objective of this study was to determine the physicochemical composition of goat milk collected from different farms and evaluate the relation of feedstuffs nutrient on the milk quality using Principal Component Analysis (PCA).

## MATERIALS AND METHODS

### Sample Collection

Goat milk samples were collected from eight farms raising Saanen (Saanen pure, Saanen cross, and Saanen-Boer cross) and Jamnapari (Jamnapari Koplo, Jamnapari Peranakan Etawa cross, Jamnapari-Toggenburg cross, and Jamnapari cross) goats indoors, in Negeri Sembilan and Selangor, Malaysia. Milk samples collected were from the type of dairy breed Saanen, dual-purpose (meat and milk) breed Jamnapari, and crossbreed goats. At each farm, milk from 5 goats in their second to fourth lactation month, were collected during their morning milk time (between 7am to 9am), stored at 4°C in an icebox and brought to the laboratory in USIM, Nilai, Negeri Sembilan. The milk samples from each farm were divided into few portions then immediately stored at −20°C until further analyses. The types of forage, animal feed, and supplements used in each farm were carefully recorded and samples collected for analysis. The respective farm locations, goat breeds, and feed types are listed in Table 1.

### Sample Preparation

A portion of the milk sample collected was freeze dried to turn to solid form for use in proximate analysis of fat and ash content. The milk sample that was frozen in freeze drying flask at –20°C for 24 h was freeze dried using Labconco FreeZone 4.5 L Freeze Dry System (Missouri, USA) with auto refrigeration mode and vacuum set to 0.770 mbar until sample completely dried. The weight of flask and sample were recorded before and after freeze drying process. Dried sample was then stored in airtight container, labelled, and placed in a chiller. The mean difference of milk solid content between the oven and freeze drying method was 0.12% ± 0.10 (*p* > 0.05).

### Total Solid Content and Proximate Analysis of Milk

Goat milk total solid content (TSC) was determined by the gravimetric method according to the method of IS 12333-1997/ISO 6731:1989 (Bureau of Indian Standards 1997). Percentage of moisture content of milk was determined by the difference between hundred and the determined percentage of TSC.

The fat and ash proximate compositions of goat milk were carried out on freeze dried samples while that of protein was performed using the liquid samples. Total fat content was determined using a Soxtherm (Gerhardt, Germany) automatic system according to the method of AOAC (2005). Total N content of milk samples was determined using a Kjeldatherm (Gerhardt, Germany) digestion system followed by automatic distillation and titration using a Vapodest50s (Gerhardt, Germany) according to the method of AOAC (2005). Non-protein N was analysed in samples of milk filtrate after precipitation with 12% (w/v) trichloroacetic acid (Friendemann Schmidt Chemical, Western Australia) according to the method of ISO 8968-4 (2001). Protein content of milk was calculated as the difference between total N and non-protein N as described by Molina-Alcaide *et al*. (2010) and final values of N were converted to the corresponding protein by a factor of 6.38 (AOAC 2005). Total ash was determined using a muffle furnace (Carbolite, United Kingdom) at 550°C according to AOAC (2005) method. Total carbohydrate content of milk was calculated as the difference between the amount of milk total solid and the sum of fat, protein, and total ash. Analyses were performed in triplicate and results were expressed as wet weight basis.

### Analysis of Feedstuffs Composition

Dry matter (DM) of feed samples was determined according to the method described by Undersander *et al*. (1993). Analysis of ether extract (crude fat), crude protein (CP), crude fibre (CF), and total ash was carried out according to the AOAC (2005) method and percentages of the nutrients were calculated following the formulas stated by Undersander *et al*. (1993) for forage analyses. Ether extract (EE) of feed samples was determined by petroleum ether extraction using a Soxtherm followed by evaporation to a constant weight. Total N content was determined using Kjeldatherm and Vapodest 50s instruments then, N values were converted to crude protein by multiplying by a factor of 6.25 (AOAC 2005; Undersander *et al*. 1993). Crude fibre of feed samples was analysed using a Fibertherm (Gerhardt, Germany) instrument programmed with two washing phases include 0.13 M sulphuric acid and 0.313 M sodium hydroxide solution (R&M Chemicals, United Kingdom). The total ash content was evaluated by dry ashing at 550°C in a muffle furnace. Analyses were performed in triplicate and results were expressed as dry weight basis.

### Statistical Analysis

The data obtained were statistically analysed using Minitab 16 software program for analysis of variance, one-way ANOVA and the mean differences were determined using Tukey’s range test with the level of statistical significance set at *p* < 0.05.

Principal component analysis using The Unscrambler X10.3 software (CAMO Software, Norway) was performed on the data in order to visualise the underlying data structure. The PCA is one of the tools for data analysis which used to gather an overview and identify patterns in the data as well as expressing the data in such a way as to emphasise their similarities and differences (Benincasa *et al*. 2008; Shin *et al*. 2010). Shin *et al*. (2010) stated that the PCA can compress the data by reducing the dimensionality of the data without much loss of information based on their similarities and differences, and define a limited number of principal components (PC) which describe independent variation structure in the data. According to Oliveira *et al*. (2014), the first principal component (PC1) is determined in the direction of greatest data variance while second principal component (PC2) is defined to be orthogonal to PC1 and represents the maximum variance not explained by PC1. The remaining PCs are obtained in the same way in decreasing order of variance. In the PCA model, samples that are close to each other present similarities and samples that are projected on the same side with variables in the plot are considered to have high values of those variables. Benincasa *et al*. (2008) stated that the absolute value of the loading (variable) in a component (between 0 and 1) describes the importance of its contribution to the variation of the PCA model generated. The variables that are far away from the origin contribute the most variation to the principal component. Besides, variables that are near each other in the loading plot are positively correlated whereas variables that opposite to each other are negatively correlated (Benincasa *et al*. 2008).

## RESULTS AND DISCUSSION

### Total Solid Content and Proximate Composition of Goat Milk

The physicochemical composition of milk from different farms is shown in Table 2. Milk moisture content ranged from 82.46% to 89.05% and TSC was between 10.95% to 17.54%. All farms raising Jamnapari-type goats except the Jamnapari-Toggenburg cross from farm 7 (F7-JTC), had significantly higher (*p* < 0.05) total solid, fat, and protein content compared to all Saanen-type goats. The milk of F7-JTC contained significantly highest (*p* < 0.05) moisture content among the Jamnapari-type goats similar to the other Saanen-type goats. Milk produced from Saanen-Boer cross from farm 4 (F4-SBC) had significantly lower moisture content (*p* < 0.05) compared to the other Saanen-type goats. The trend of similarity in the total solid and protein content was also observed between milk from F7-JTC with other Saanen-type goats except for F4-SBC. Results of the present study on TSC of goat milk from Saanen-type goats were within the range reported by Žan *et al*. (2006) and Sung *et al*. (1999) but the TSC of Jamnapari-type goats were much higher than those reported by Ramadhan *et al*. (2013) and Singh *et al*. (2014). The low TSC content of Saanen milk is consistent with its high moisture content and characteristic of Saanen as dairy goats (Mayer & Fiechter 2012; Almeida *et al*. 2013).

The content of fat in milk from the farms ranged from 2.49% to 7.36%. Milk from farm 5 with Jamnapari Koplo goats (F5-JK) had the highest fat content followed by farm 6 (Jamnapari Peranakan Etawa cross, F6-JPEC) and farm 8 (Jamnapari cross, F8-JC). The results were higher than reported by Singh *et al*. (2014) for Jamnapari goats (4.61% to 5.17%) and Ramadhan *et al*. (2013) for Etawa goats (5.98% to 6.98%). However, milk fat from F7-JTC showed lower value than the Jamnapari-type goats that reported by Singh *et al*. (2014) and Ramadhan *et al*. (2013). There were no significant differences (*p* > 0.05) between fat content of all Saanen-type goats except from F4-SBC milk. Percentage of milk fat for Saanen-type goats were in the range reported by Žan *et al*. (2006) (2.28% to 6.20%), but lower compared to Mayer and Fiechter (2012) (3.73% ± 0.46), da Costa *et al*. (2014) (3.55% ± 0.21) and Razzaghi *et al*. (2015) (3.36% to 3.57%) of the different locations.

Protein content in all milk samples ranged between 3.10% to 6.19%. Results of milk protein for the farms with Saanen-type goats were consistent with those reported by Sung *et al*. (1999) and Žan *et al*. (2006) but higher than values reported by Razzaghi *et al*. (2015). In the case of Jamnapari-type goats, milk protein of F7-JTC was similar to that reported by Singh *et al*. (2014) while that of F5-JK, F6-JPEC, and F8-JC were higher than those reported by Agnihotri and Prasad (1993) and Hassan *et al*. (2010). Differences in milk constituents compared to the literature can be attributed to several factors such as season (Morand-Fehr *et al*. 2007; Chen *et al*. 2014), lactation stage (Agnihotri & Rajkumar 2007; Singh *et al*. 2014), parity of goat (Agnihotri & Rajkumar 2007; Goetsch *et al*. 2011) as well as quality and level of nutrient in the feed ration (Sanz Sampelayo *et al*. 2007; Molina-Alcaide *et al*. 2010).

Total ash and total carbohydrate content of the milk varied considerably, regardless of goat breeds. Milk ash content ranged from 0.67% to 0.86% and was in agreement with that reported by Mahmood and Usman (2010) (0.56% to 0.99%). The milk carbohydrate values were between 3.26% to 4.71% and were in the range with the findings of Žan *et al*. (2006) and Mayer and Fiechter (2012) but were lower than reported by Almeida *et al*. (2013) and Hassan *et al*. (2010).

**Figure 1:**
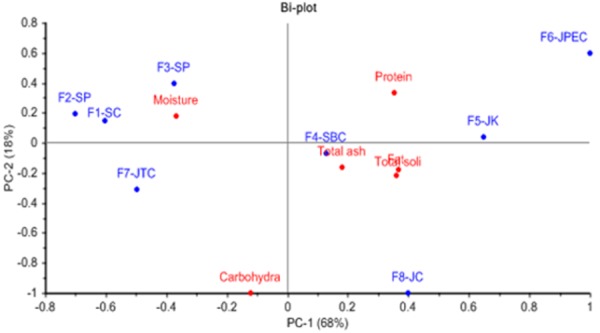
PCA model of milk composition.

Principal component analysis was used to determine the correlation between the nutrients in milk (dos Santos *et al*. 2013). By using the milk composition data from Table 2, the PCA model of milk composition was plotted as shown in Figure 1. The PCA model shows the distribution pattern of samples from the different farms and the milk compositions in the first two principal components. The first two PCs (PC1 and PC2) showed 86% of the variances in the data set. The PC1 accounted for 68% of the total variation in the data contributed mainly by four variables namely total solid, fat, protein, and moisture content. All these variables were positively correlated, except moisture content which was located at the negative loading of PC1. The PC2 accounted for 18% of the total variance, with carbohydrate as the dominant variable.

Position of samples in the positive quadrant of PC1 showed milk from F5-JK and F6-JPEC goats contained the highest total solid, fat, and protein content compared to other farms. In contrast, among the Jamnapari breed, milk from F8-JC goats had high carbohydrate and total ash in addition to high total solid and fat. Milk from F4- SBC goats had high values of total ash, total solid, and fat content. The negative quadrant of PC1 showed milk from F1-SC, F2-SP, and F3-SP which raised Saanen pure and crossbreed goats contained high values of moisture but were low in total solid, fat, and protein content. Further, milk from F7-JTC goats differed from the other Jamnapari breeds and contained high moisture and carbohydrate content but low total solid, fat, and protein values. These differences in milk composition could be contributed by the different types of goat breed. For instance, crossing Saanen with Boer which is a meat producing breed, could explain the variation in milk composition while, the milk from F7-JTC which raised Jamnapari crossed with Toggenburg, a milk producing breed, produced milk that closely resembled that of the Saanen breed. Mayer and Fiechter (2012) reported that despite considerable seasonal variations, there was no statistical significant differences in milk chemical composition between the six dairy goat breeds include Saanen, Toggenburg, White, Strahlen, Coloured, and Pinzgau (11.93% to 12.47% total solid, 3.51% to 3.86% fat, 3.29% to 3.44% protein, and 0.819% to 0.843% ash). Park (2010) also recorded that dairy breeds include Toggenburg, Alpine, Oberhasli, and LaMancha produce milk yield and composition in between the Saanen and Nubian breed.

### Chemical Composition of Feedstuffs and its Relation to Milk Quality

**Figure 2:**
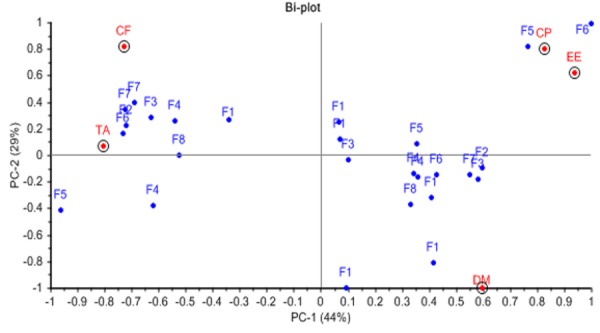
PCA model for chemical composition of the different feedstuff types.

Differences in composition of milk samples between farms might be due to genetic variation between type of Saanen and Jamnapari breeds while differences within the similar breeds could be contributed by factors such as feed or diet given to goats. In this study, the feedstuffs available for goats at each farm were analysed for its nutrient composition to observe the relationship between the feedstuffs used to the milk quality. Table 3 shows the average chemical composition of the different feedstuff types and the data were used to generate the PCA model that shown in Figure 2. Figure 2 shows the distribution pattern of the feedstuffs nutrients of the different farms. The first two PCs (PC1 and PC2) showed 73% of the variances in the data set. The PC1 accounted for 44% of the total variation in the data contributed mainly by three variables namely crude protein, ether extract, and crude fibre content while, PC2 accounted for 29% of the total variance, with dry matter as the dominant variable.

Analysis showed that the high concentration of fat in F5-JK and F6-JPEC milk could be due to the diet containing high EE or crude fat content given by the respective farms (Fig. 2; points located farthest in positive quadrant of PC 1). The high EE content in diet was contributed by feedstuffs soy-bran mixture and soy waste which contained 6.53% and 10.39% EE, respectively (Table 3). Other than the green forage used as a source of fibre in goat feed, the soy-bran mixture and soy waste each contained high CF content of 27.36% and 25.66%, respectively.

According to Cannas (2004) and Zenou and Miron (2005), inclusion of soybean hulls in goats feed increased milk yield and milk fat concentration due to the high content of digestible fibre in the hulls. It is suggested that digestible fibre increases the acetic acid availability for milk fat synthesis and stimulates energy partitioning towards milk synthesis instead of body fat reserve deposition (Sanz Sampelayo *et al*. 2007). Sources of fibre used by farmers in this study include Napier grass, barley sprouts, rice straw, mix grasses, and Sarang Buaya (Ischaemum timorense Kunth) grass. Fibre content of Napier grass (36.21%) in F6-JPEC may also contribute to the high milk fat value. Thus, this indicates that a mixture of feed with high lipid and fibre content may lead to the increased of milk fat content.

The PCA model also shows a cluster of feedstuffs of the different farms plotted in the negative quadrant of PC1. The feedstuffs were mainly from the type of forages of grasses variety which contained high CF and TA content but low in DM (Table 3). Next, the cluster of feedstuffs plotted in the positive quadrant of PC1 showed feedstuffs samples that contained high DM in addition to high EE and CP content. These feedstuffs were from the type of concentrate feeds include feed pellets and brans which had DM ranged from 87.23% to 90.57%, 3.35% to 5.64% EE and 12.95% to 24.72% CP content (Table 3). According to Morand-Fehr *et al*. (2007), concentrate feeds were given to animals both in outdoor and indoor farming systems with a variety of forage to concentrate ratios in the feed ration to compensate the dietary nutrient required by the animal as well as to manipulate the composition and quality of the ruminant milk. Similarly, the local farmers may also use the concentrates to support the dietary nutrient in the feed rations thus contribute to the variation of the milk composition. While, the differences in nutrient content of the pellets and brans were due to the different ingredients or raw materials of commercial concentrates produced by the manufacturers.

Besides, the high milk protein content in F5-JK and F6-JPEC might also be contributed by the high CP in the feedstuffs of both farms (Fig. 2; points located farthest in positive quadrant of PC 1). Soy-bran mixture and soy waste used in the farms showed the highest CP content compared to the other feed types, 38.57% and 35.08%, respectively. Further, the inclusion of different feed types like foliage and concentrates including feed pellets and brans that are high in CP might influence the milk protein concentration. According to Zervas *et al*. (1998), protein content was significantly (*p* < 0.001) increased in ewes milk when soybean hulls were used as a replacement for maize in concentrate diet. However, Cannas *et al*. (1998) reported that substitution of concentrate in feed ration with soybean hulls and beet pulps decreased the protein concentration in ewes milk while, Vasta *et al*. (2008) stated that the decreased in milk protein concentration observed with the use of soybean hulls and beet pulps was probably due to a dilution effect. Morand-Fehr *et al*. (1991) also reported that the protein and casein contents do not appear to be particularly sensitive to changes in the protein source although the goats were given isoenergetic and isonitrogenous diets. In contrast, Goetsch *et al*. (2011) stated that the effect of dietary CP level on the composition of milk depends on the nature of nitrogenous compounds in the feed as it will influence the metabolisable protein intake. Besides the differences in type, level, or dietary nutrient of feedstuffs in a feed ration contribute to the variation of milk composition, the effectiveness was affected also by factors including DM and nutrient intakes (Molina-Alcaide *et al*. 2010; Steinshamn *et al*. 2014), nutrient degradability (Sanz Sampelayo *et al*. 1999; Morand-Fehr *et al*. 2007), and nutrient digestibility (Gwayumba *et al*. 2002; Molina-Alcaide *et al*. 2010; Kholif *et al*. 2014) in the ruminant.

Farms F2-SP, F3-SP, F6-JPEC, and F7-JTC showed high ash content in the feed composition (Fig. 2; close to total ash point) however, the trend of ash content in milk did not reflect the dietary intake. Agnihotri and Rajkumar (2007) found that although ash content of goat milk ranged between 0.76% to 1.11% for different varieties of goat breeds, these values were not significantly different. Mech *et al*. (2008) reported that ash content was found to be the least variable milk constituent and did not vary significantly among different lactation stage. However, a different result was reported by Voutsinas *et al*. (1990) which recorded that ash content progressively increased as lactation proceeded. Although total ash shows least variation, the specific constituents of ash like minerals and trace elements may vary due to factors including environmental condition, animal feed and nutrition, lactation stage, animal species or breed, and contaminants (Zain *et al*. 2016; Singh *et al*. 2015).

In this study, there was variation of milk total carbohydrate in which lactose is considered as the main milk carbohydrate (Aplocina & Spruzs 2012; Sanz Ceballos *et al*. 2009), regardless of the different goat and feed types among farms. According to Aghsaghali and Fathi (2012), high lactose production is accompanied by high milk volume production whereby this could be seen in goats of dairy breed which produces high milk yield (Aghsaghali & Fathi 2012). However, the milk lactose content is also affected by the level of blood glucose in ruminant as glucose is the main precursor for the synthesis of lactose in mammary gland and epithelial cells (Bauman & Currie 1980; Aghsaghali & Fathi 2012). In the study of goat in South Africa, Pambu (2011) reported that indigenous goat breed had higher lactose content (average of 8 weeks, 4.6%) than dairy goat breeds (4.1%) because indigenous does had higher blood glucose concentration than the dairy does. Besides, Molina-Alcaide *et al*. (2010), Almeida *et al*. (2013) and Razzaghi *et al*. (2015) found no significant differences in the content of milk lactose although goats were fed with different dietary treatments and there was only a low tendency (0.05 < P < 0.10) for the differences in lactose yield (Razzaghi *et al*. 2015). Therefore, it could be suggested that the different lactose content in milk of the different goat breeds might be due to the genetic variation in manipulating the glucose source in lactose production mechanisms and the differences were less influenced by the dietary factor.

## CONCLUSION

The physicochemical composition of goat milk differed among the different farms. Data from the analysis of milk composition and PCA model showed that milk of Jamnapari-type goats from F5-JK, F6-JPEC, and F8-JC had higher total solid, fat, and protein content compared to the Saanen-types goat. While, the physical and chemical composition of milk from Jamnapari-types goat from F7-JTC was much similar to the other Saanen-type goats and this might due to the crosses with dairy breed which its moisture content was much higher than the solid content. Besides the genetic variation between goat breeds, the feeding of feedstuffs which high in lipid and fibre content, for instance, the soybean-based products may lead to the increased of fat content in the goat milk.

## Figures and Tables

**Table 1:** Goat breeds and feed types from different farms.

Farm location	Goat breed	Feed type
Farm 1 (F1), Hulu Langat, Selangor	Saanen cross (SC)	Napier grass silage Barley sprouts Dates Mulberry leaves Corn grain Goat pellet
Farm 2 (F2), Banting, Selangor	Saanen pure (SP)	Napier grass fresh Dairy bran
Farm 3 (F3), Hulu Langat, Selangor	Saanen pure (SP)	Napier grass fresh Goat pellet 1 Goat pellet 2
Farm 4 (F4), Sungai Buloh, Selangor	Saanen–Boer cross (SBC)	Napier grass fresh Grower pellet Breeder pellet Rice straw
Farm 5 (F5), Nilai, Negeri Sembilan	Jamnapari Koplo (JK)	Napier grass silage Wide leaves Soy-bran mixture
Farm 6 (F6), Sungai Buloh, Selangor	Jamnapari Peranakan Etawa cross (JPEC)	Napier grass fresh Soy waste Goat pellet
Farm 7 (F7), Shah Alam, Selangor	Jamnapari–Toggenburg cross (JTC)	Napier grass fresh Mix grasses Dairy bran
Farm 8 (F8), Jeram, Selangor	Jamnapari cross (JC)	Sarang Buaya grass (*Ischaemum timorense* Kunth) Mix bran

**Table 2:** Physicochemical composition of milk from different farms (% wet basis).

Composition (%)	Farms*							

	F1-SC	F2-SP	F3-SP	F4-SBC	F5-JK	F6-JPEC	F7-JTC	F8-JC
xMoisture content	89.05^a^± 0.13	88.94^a^± 0.05	88.87^b^± 0.02	85.37^d^± 0.03	82.84^f^± 0.01	82.46^g^± 0.03	88.19^c^± 0.01	83.60^e^± 0.01
Total solid content	10.95^g^± 0.13	11.06^f^± 0.05	11.13^f^± 0.02	14.63^d^± 0.03	17.16^b^± 0.01	17.54^a^± 0.03	11.81^e^± 0.01	16.40^c^± 0.01
Fat	2.49^e^± 0.04	2.78^e^± 0.19	2.89^e^± 0.39	5.49^c^± 0.18	7.36^a^± 0.02	7.14^a^± 0.05	3.62^d^± 0.02	6.59^b^± 0.03
Protein	3.58^de^± 0.11	3.39^e^± 0.02	3.71^d^± 0.02	4.34^c^± 0.06	5.08^b^± 0.18	6.19^a^± 0.13	3.10^f^± 0.02	4.23^c^± 0.04
Total ash	0.76^c^± 0.001	0.67^d^± 0.01	0.86^a^± 0.02	0.77^bc^± 0.001	0.76^c^± 0.003	0.85^a^± 0.01	0.79^b^± 0.003	0.86^a^± 0.003
Total carbohydrate	4.01^bc^± 0.15	4.01^bc^± 0.25	3.64^cd^± 0.41	4.00^bc^± 0.26	3.79^bc^± 0.20	3.26^d^± 0.07	4.29^ab^± 0.03	4.71^a^± 0.05

* F1-SC = Farm 1 with Saanen cross, F2-SP = Farm 2 with Saanen pure, F3-SP = Farm 3 with Saanen pure, F4- SBC = Farm 4 with Saanen-Boer cross, F5-JK = Farm 5 with Jamnapari Koplo, F6-JPEC = Farm 6 with Jamnapari Peranakan Etawa cross, F7-JTC = Farm 7 with Jamnapari-Toggenburg cross, F8-JC = Farm 8 with Jamnapari cross goats.

Mean ± standard deviation

Means in the same row with different superscripts are significantly different (*p* < 0.05)

**Table 3:** Chemical composition of feedstuffs from different farms (% dry basis).

Farm	Feed type	Composition (%)				

		Dry matter	Crude protein	Ether extract	Crude fibre	Total ash
Farm 1	Napier silage	20.12 ± 0.04	11.26 ± 0.33	2.92 ± 0.02	39.76 ± 1.29	5.20 ± 0.14
	Barley sprout	14.17 ± 1.44	21.36 ± 2.81	2.44 ± 0.27	24.84 ± 7.08	3.39 ± 0.56
	Dates	80.36 ± 0.35	2.53 ± 0.10	0.17 ± 0.09	2.62 ± 0.34	1.70 ± 0.17
	Mulberry leaves	36.30 ± 0.01	33.07 ± 2.40	1.24 ± 0.15	12.38 ± 0.17	12.13 ± 0.36
	Corn grain	87.54 ± 0.36	10.14 ± 0.30	1.61 ± 0.07	3.05 ± 0.40	1.23 ± 0.16
	Goat pellet	90.15 ± 0.04	19.65 ± 0.20	4.13 ± 0.07	12.97 ± 0.44	8.39 ± 0.11
Farm 2	Napier fresh	15.12 ± 0.01	12.05 ± 0.20	1.45 ± 0.38	37.46 ± 0.72	11.70 ± 0.36
	Dairy bran	88.45 ± 0.03	24.72 ± 1.15	5.64 ± 0.28	14.40 ± 1.03	8.89 ± 0.41
Farm 3	Napier fresh	13.42 ± 0.04	14.66 ± 0.85	1.41 ± 0.22	37.98 ± 0.33	10.40 ± 0.25
	Goat pellet	1 87.66 ± 0.04	12.95 ± 1.76	4.58 ± 0.33	39.25 ± 0.70	5.15 ± 0.16
	Goat pellet	2 88.61 ± 0.02	24.52 ± 0.68	4.70 ± 0.26	13.37 ± 0.48	7.55 ± 0.46
Farm 4	Napier fresh	15.18 ± 0.03	16.40 ± 1.34	1.97 ± 0.42	32.05 ± 0.68	12.26 ± 0.67
	Grower pellet	90.57 ± 0.06	17.81 ± 0.88	5.15 ± 1.24	23.92 ± 1.72	7.90 ± 0.22
	Breeder pellet	90.23 ± 0.11	18.96 ± 0.30	4.77 ± 0.56	22.31 ± 0.80	7.76 ± 0.05
	Rice straw	86.20 ± 0.12	6.19 ± 0.16	0.90 ± 0.24	37.38 ± 0.49	11.83 ± 0.20
Farm 5	Napier silage	42.32 ± 0.15	4.78 ± 0.16	0.42 ± 0.17	17.14 ± 0.81	20.46 ± 0.44
	Wide leaves	38.50 ± 0.06	22.52 ± 0.47	4.01 ± 0.41	15.54 ± 0.32	5.42 ± 0.18
	Soy-bran mixture	20.77 ± 0.14	38.57 ± 0.66	6.53 ± 1.06	27.36 ± 1.93	3.34 ± 0.07
Farm 6	Napier fresh	15.75 ± 0.01	9.65 ± 0.42	1.70 ± 0.12	36.21 ± 0.48	11.84 ± 0.11
	Soy waste	14.68 ± 0.01	35.08 ± 0.33	10.39 ±0.65	25.66 ± 2.03	4.19 ± 0.14
	Goat pellet	88.37 ± 0.14	19.14 ± 0.35	5.39 ± 0.27	19.71 ± 1.15	8.08 ± 0.37
Farm 7	Napier fresh	13.48 ± 0.01	12.77 ± 0.47	2.52 ± 0.18	43.53 ± 0.41	11.60 ± 0.04
	Mix grasses	16.22 ± 0.07	13.04 ± 0.74	1.48 ± 0.46	45.45 ± 2.02	10.02 ± 0.18
	Dairy bran	89.77 ± 0.28	21.22 ± 1.09	5.28 ± 0.68	18.20 ± 1.20	6.51 ± 0.13
Farm 8	Sarang Buaya grass	30.49 ± 0.04	8.52 ± 0.25	1.56 ± 0.13	34.28 ± 0.74	8.01 ± 0.09
	Mix bran	87.23 ± 0.09	16.21 ± 0.31	3.35 ± 0.05	16.56 ± 1.52	5.60 ± 0.17

Mean ± Standard deviation
